# Subcellular Localization of Copper—Cellular Bioimaging with Focus on Neurological Disorders

**DOI:** 10.3390/ijms21072341

**Published:** 2020-03-28

**Authors:** Barbara Witt, Dirk Schaumlöffel, Tanja Schwerdtle

**Affiliations:** 1 Institute of Nutritional Science, University of Potsdam, Arthur-Scheunert-Allee 114–116, 14558 Nuthetal, Germany; taschwer@uni-potsdam.de; 2 Institut des Sciences Analytiques et de Physico-Chimie pour l’Environnement et les Matériaux (IPREM), UMR 5254, CNRS/Université de Pau et des Pays de l’Adour/E2S UPPA, 64000 Pau, France; dirk.schaumloeffel@univ-pau.fr; 3 TraceAge—DFG Research Unit on Interactions of Essential Trace Elements in Healthy and Diseased Elderly (FOR 2558), Potsdam-Berlin-Jena, Germany

**Keywords:** copper, cellular bioimaging, neurodegenerative diseases, copper-related disorders, SIMS techniques, TEM, S-XRF

## Abstract

As an essential trace element, copper plays a pivotal role in physiological body functions. In fact, dysregulated copper homeostasis has been clearly linked to neurological disorders including Wilson and Alzheimer’s disease. Such neurodegenerative diseases are associated with progressive loss of neurons and thus impaired brain functions. However, the underlying mechanisms are not fully understood. Characterization of the element species and their subcellular localization is of great importance to uncover cellular mechanisms. Recent research activities focus on the question of how copper contributes to the pathological findings. Cellular bioimaging of copper is an essential key to accomplish this objective. Besides information on the spatial distribution and chemical properties of copper, other essential trace elements can be localized in parallel. Highly sensitive and high spatial resolution techniques such as LA-ICP-MS, TEM-EDS, S-XRF and NanoSIMS are required for elemental mapping on subcellular level. This review summarizes state-of-the-art techniques in the field of bioimaging. Their strengths and limitations will be discussed with particular focus on potential applications for the elucidation of copper-related diseases. Based on such investigations, further information on cellular processes and mechanisms can be derived under physiological and pathological conditions. Bioimaging studies might enable the clarification of the role of copper in the context of neurodegenerative diseases and provide an important basis to develop therapeutic strategies for reduction or even prevention of copper-related disorders and their pathological consequences.

## 1. Physiology of Copper

### 1.1. General Physiological Aspects

Copper is an essential trace element present in the human body at overall levels around 90–110 mg, mainly allocated in liver, muscle and brain [1,2,3]. This trace element plays a fundamental role as cofactor of many enzymes related to energy metabolism and protection against oxidative stress. Thus, it is involved in diverse physiological pathways [4,5]. Thereby, the copper status needs to be strictly regulated since any homeostatic imbalance can have severely detrimental consequences. Both deficiency (e.g., [6,7]) and overload (e.g., [8,9]) of copper have been linked to human diseases.

In the brain, copper is of great importance for neuronal function, since it is involved in biological processes including neurotransmitter synthesis and respiratory oxidation [4,10,11,12]. Moreover, copper is not only important for basic physiological function of brain cells. Studies with primary neurons attributed neuromodulatory aspects to copper. Copper is supposed to be involved in the communication and neuronal signaling pathways [4,13,14]. Indeed, neurons release copper into the synaptic cleft in response to depolarization, leading to local concentrations up to 250 µM [15]. For detailed information on how copper is connected to synaptic transmission, refer to other reviews including [13].

### 1.2. Copper Metabolism and Pathways

Natural sources of copper are, among others, meat, vegetables and cereals, but exposure also derives from industrial application [1]. Copper absorption occurs in the small intestine, mainly in the duodenum, with an efficiency of up to 60% [16]. Via the portal vein, copper is transferred to the liver and further distributed via the bloodstream throughout the entire organism including muscles and brain. In the serum, approximately 90% of the copper is bound to the copper-binding protein ceruloplasmin. Besides, copper is also bound to albumin and transcuprein [17,18,19]. Copper in its free form can be potentially harmful due to its high chemical redox activity. To prevent uncontrolled redox activity under physiological conditions, copper is almost exclusively associated with transport proteins and its free levels are maintained at low levels (10^−18^ M; amounts to less than one free copper ion per cell) [20,21].

Copper plays a pivotal role for the neuronal functions and is delivered to the brain under strictly controlled conditions. Transfer and thus regulation of copper homeostasis in the brain occurs via the blood–brain barrier (BBB) and blood–liquor barrier (BLB) [13,22]. While BBB seems to be the major route for copper uptake from blood circulation into the brain, where copper is further released to the cerebrospinal fluid (CSF), BLB is discussed to be rather involved in the secretory pathway of copper from CSF back into the blood circulation. In that way, the homeostasis of copper under physiological conditions is maintained in the brain [4,22].

Within the brain, copper seems to be unevenly distributed—not only among the different brain regions (highest copper found in *locus coeruleus* and *substantia nigra* [5]), but also among the specific brain cells. Histochemical studies with brain slices revealed that glial cells show higher copper levels compared to neurons, interestingly, under both physiological and pathological conditions [4,5,23]. Furthermore, both, the total copper level in the brain and its spatial distribution are altered in neurological disorders including Wilson and Alzheimer’s disease [4,24,25].

### 1.3. Cellular Distribution of Copper

It is still the focus of investigation how copper is taken up and distributed within the cells. This paragraph gives a brief outline of the current state of research on copper homeostasis at the cellular level, its regulation and the biochemical pathway throughout the cell.

#### 1.3.1. General Cellular Copper Homeostasis

**Figure 1:**
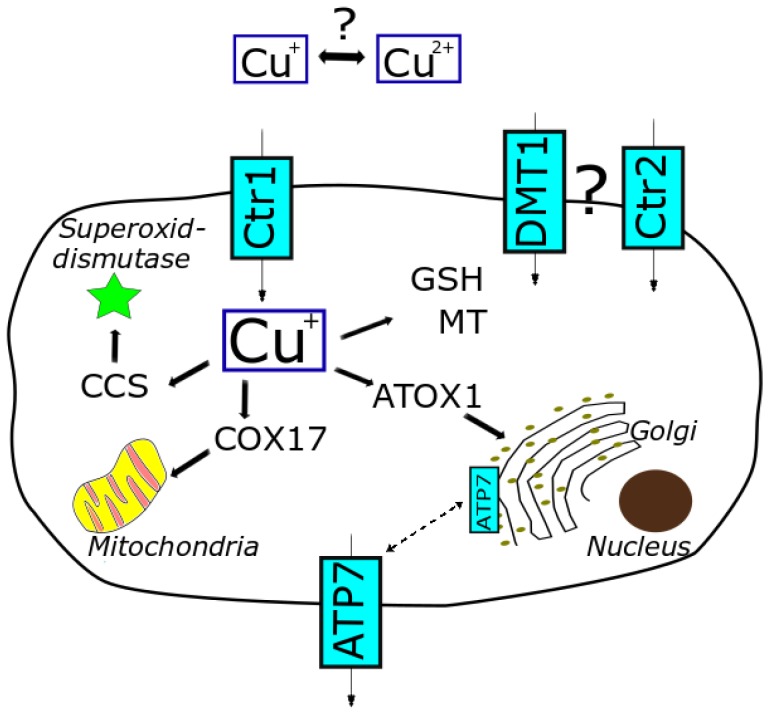
Cellular copper homeostasis, relevant transporters and proteins involved in distribution and trafficking of copper (based on [8,10,30]).

In the peripheral tissue, cellular uptake of copper mainly occurs via the high-affinity copper transport receptor 1 (Ctr1) [26]. The low-affinity copper transport receptor 2 (Ctr2), the divalent metal ion transporter (DMT1) and the anion exchange transporter might be also involved [27]. Copper is transferred across the cell membrane and taken up as Cu(I). Therefore, Cu(II) needs to be extracellularly reduced to Cu(I) by cuprireductases. However, the exact site and mode of action still remain the focus of investigation [21,28]. Cu(II) is also discussed to be taken up by the cells, yet the underlying mechanisms are less understood. Important cellular efflux transporters are the proteins ATP7A and ATP7B [18,29]. Consequently, copper homeostasis is maintained by tight regulation of cellular uptake, distribution and excretion via transporters and copper-binding proteins (storage and escorting) (see Figure 1) [19].

Within the cell, copper is mainly sequestered by metallothionein (MT) and glutathione (GSH) to keep free copper at a low level. Following uptake into the cell, Cu(I) is supposed to be directly transferred from the uptake transporter Ctr1 via GSH to further shuttle proteins, thereby avoiding dissociation as free copper [21]. These shuttle proteins include chaperones such as human antioxidant protein-1 (ATOX-1), copper chaperone for superoxide dismutase 1 (CCS) and cytochrome c-oxidase (COX17). These chaperones play an important role in capturing free copper and trafficking copper throughout the cell [17]. In that way, the level of free cellular copper is highly limited and an adequate balance between essential supply and prevention of potential toxicity is ensured. Moreover, these chaperones are not restricted to just escorting copper to different subcellular compartments but gained focus playing a decisive role in physiological pathways [21].

An essential regulator of the homeostatic process is the copper-dependent localization [21]. Depending on the copper availability and status of cellular copper level, the uptake transporter Ctr1 is shuttled to or away from the cell membrane. This re-localization process regulates the cellular copper balance. By this means, the cell is protected against increasing copper levels. Any dysregulation of these mechanisms might lead to adverse alterations in the cellular copper level and spatial allocation, which are important hallmarks of copper-related diseases (e.g., Menkes, Wilson). In fact, changes of subcellular copper distribution have been detected in Menkes disease patients. Here, cellular copper was mainly accumulated in the cytosol, nuclei and mitochondria [18]. Moreover, even though overall copper levels in the liver of diseased and non-diseased animals were not significantly different, observed liver damage correlated with the overload of copper in the mitochondria in connection with impaired mitochondrial function in the diseased animals [31,32]. This context illustrates the importance not only to assess total copper levels, but also to localize the distribution within the single organelles on a subcellular level.

Indeed, mitochondria play an important role in the copper homeostasis. On the one hand, mitochondria need an adequate copper supply to maintain their physiological function (superoxide dismutase, cytochrome c-oxidase). On the other hand, they are discussed to act as important copper storage [32,33]. Thereby, copper is shuttled to the mitochondria most likely by copper chaperones including COX17. The majority of mitochondrial copper seems to be stored as Cu(I)-anionic complex in the matrix; however, the ligand still is not exactly identified [34,35,36]. In order to use this pool for the synthesis of cuproenzymes, copper needs to be redistributed to other cellular sites. Thus, mitochondria seem to provide a distinct storage capacity and might act as a buffering system in case of copper overload, though only within certain limitations. Disorders including Wilson disease point out these limits, where high levels of mitochondrial copper induce both structural and functional alterations of the mitochondria [35]. Mitochondrial destruction following copper overload is supposed to be a quite sensitive marker since structural changes and functional abnormalities in the mitochondria are sensed already at an early stage [32,37]. Therefore, assessing mitochondrial characteristics and functions might serve as a powerful diagnostic biomarker for copper-related diseases including Wilson disease [31,32].

Besides mitochondria, the Golgi apparatus also provides a copper pool, which is important to process copper-dependent proteins. Copper is delivered to this network by the chaperone ATOX1 and the transporters ATP7A and ATP7B. Moreover, these transporters are involved in the regulation of cellular redistribution and finally secretion of copper. In response to high intracellular copper concentrations, ATP7A and ATP7B can translocate from Golgi network to the plasma membrane and thus shuttle copper out of the cell [34]. Indeed, copper-dependent re-localization here contributes as well to maintain the copper homeostasis and to restore physiological conditions.

#### 1.3.2. Cellular Copper Homeostasis in the Brain

There are still open questions concerning the balance of copper homeostasis within the brain, which is supposed to be particularly vulnerable to any alterations. However, it seems apparent that many of the mentioned key players involved in the regulation of peripheral copper balance are also relevant in the brain [4,13,22,28,38]. Besides the above-discussed mechanisms to maintain copper homeostasis, further proteins are discussed to be part of this complex regulatory system in the brain. One such copper-binding glycoprotein, predominantly expressed in brain cells, is the prion protein (PrP). The expression of PrP is dependent on the extracellular copper levels. It seems that PrP is released by the neurons in response to high extracellular copper. Copper is bound to the released PrP and shuttled to the astrocytes, where it is stored. Thereby, astrocytes assist with the clearance of copper and keep neurons safe from copper overload [39,40,41]. Another protein in this context is the amyloid precursor protein (APP). This membrane-associated protein has been shown to be involved in physiological processes including cell growth but also transcellular transport and trafficking within the cell [42,43,44]. Besides having two copper-binding sites, it also possesses copper reductase activity. This characteristic might be responsible for enhanced copper toxicity due to free radical formation associated with Alzheimer’s disease (see Section 2.2) [44,45].

In summary, the cells evolved complex regulatory systems with orchestrated processes mediated by numbers of transporters and copper-binding proteins including chaperones. This network enables the maintenance of copper homeostasis and the regulation of the cellular distribution of copper (see Figure 1) required for appropriate functions in physiological processes.

### 1.4. Homeostasis and Connection to Other Essential Elements

The homeostasis of copper is tightly balanced and regulated. In general, an intracellular overload of copper is supposed not to be a result of dietary excess but rather a result of disturbance of the copper homeostasis [1,46]. As aforementioned, there are various mechanisms in the body to maintain and regulate the copper homeostasis. Other trace elements might also interact with this network. With regard to neurodegenerative diseases, this interplay will be exemplarily shown for the trace elements iron and zinc.

#### 1.4.1. Iron

Iron is the most abundant essential trace element in the body, being involved in many fundamental physiological processes such as oxygen transport [47,48]. Comparing both elements, it becomes apparent that iron and copper show similar physicochemical characteristics. Therefore, an interplay on the physiological level seems likely. One link is the copper-binding protein ceruloplasmin [4]. This cuproenzyme possesses ferroxidase activity. During the export process of iron, ceruloplasmin catalyzes the oxidation of iron, and thus, affinity to the iron-binding protein transferrin is ensured. Besides being an important copper-carrier, ceruloplasmin regulates the iron homeostasis and clearance from the brain and other tissues. In that way, both iron and copper metabolism are connected [49,50]. Indeed, deficiency in copper results in lowered ceruloplasmin levels and eventually deposition of iron in the brain [4,51]. In the case of Wilson disease, accumulation of both copper and iron is observed in the brain, probably also due to lower ceruloplasmin levels. Iron is moreover supposed to be involved in the formation of senile plaques in the brain associated with Alzheimer’s disease (see Section 2.2) [52,53,54].

#### 1.4.2. Zinc

Zinc is required for physiological processes particularly concerning the immune system and seems to act as a competitive opponent of copper [55,56]. Under specific physiological circumstances, certain zinc transporters have been proposed to also be involved in the copper transport leading to competition for transfer [18]. This interaction might be used against copper-related disorders mediated by copper overload. Indeed, treatment with zinc has been shown to limit in vitro copper uptake into the cell [4,56,57,58]. At the presymptomatic stage, zinc is applied for early treatment of Wilson disease. By increased uptake of zinc, the copper absorption can be reduced [50,57].

Such links between the homeostasis of elements illustrate that the single element species are not just regulated on their own. The homeostatic mechanisms are connected and build up a complex network within the scope of human physiology, and moreover, pathophysiology. There are still many open questions regarding uptake, excretion and trafficking mechanisms on the molecular level and how the different trace elements are connected under physiological conditions and in the context of brain-related disorders.

## 2. Toxicology of Copper

### 2.1. Cellular Mechanisms

The copper status needs to be tightly regulated since both deficiency and overload are critical. Different toxic modes of action have been discussed for copper toxicity. In general, excess dietary intake of copper does not seem to be the primary cause for its toxicity but rather disrupted homeostasis via dysregulated mechanisms regarding cellular absorption, distribution and excretion [1].

Excess copper levels might increase oxidative stress resulting in DNA damage and oxidation of proteins and lipids [59,60,61]. Due to their redox ability, free copper ions can induce reactive oxygen species (ROS) via Fenton-like and Haber–Weiss-based reactions. To prevent these adverse reactions under physiological conditions, copper is mainly bound to chaperons [34,46]. The relevance of oxidative stress via ROS production in the context of copper toxicity might be questioned under physiological conditions since the level of “free” unbound copper is kept at a low level. Whether copper is involved in oxidative mechanisms in vivo is not fully elucidated, though controversially discussed. To date, however, no treatment with antioxidants has been successful to counteract any adverse effects of copper [62]. Even though oxidative damage has been associated with copper toxicity in copper-overloaded cells, it does not seem to be the primary cause leading to cell death but is supposed to be rather a late state of copper-toxicity in already massively damaged cells [63]. In this context, GSH is considered to a be protective agent, though mainly due to its binding capacity for Cu(I), thus preventing interactions with other cellular proteins rather than its redox regulation [62].

Furthermore, both Cu(I) and Cu(II) show high affinity for a number of protein sites. Particularly histidine, cysteine and methionine are supposed to play important roles as ligands. By replacing the original binding metal at the active site or misfolding due to copper protein interaction, the protein function can be adversely affected [62,64].

One of the main cellular targets for copper toxicity seems to be mitochondria [65]. On the one hand, deficiency leads to adverse effects, since copper is required for fundamental physiological processes in the mitochondria. On the other hand, excess copper might destruct the mitochondria [4,41,66]. In fact, an overload of copper shows effects on the mitochondrial structure and mitochondrial proteins and thus affects their functions [31,63,67]. Especially respiratory chain complexes I, III and IV seem to be disrupted by copper and might lead to massive ROS release [63,68]. Particularly in the brain, due to its high metabolic activity, mitochondria play an essential role to maintain the physiological brain function. Therefore, the brain may be highly susceptible to copper toxicity. In fact, neurotoxic effects have been first ascribed to copper based on studies with cat brains [69]. Adverse effects in the neurons have been dedicated to functional disruption of mitochondria in connection with increased oxygen expenditure. Thereby, mitochondria were clearly linked to copper toxicity. Hence, copper exerts its toxic effects on several cellular sites with mitochondria being the most likely target organelles.

### 2.2. Copper-Related Diseases with Focus on Neurological Disorders

Homeostasis in the brain needs to be strictly maintained and regulated since imbalances might affect brain development and function. In fact, a dysregulated copper homeostasis has been associated with neurodegenerative disorders. This link will be described in the following paragraph in the context of Alzheimer’s and Wilson disease. Both diseases are associated with alteration of copper levels in the brain. In the case of Wilson disease, the copper transporters are affected due to genetic mutations, resulting in severe homeostatic disturbances. In Alzheimer’s disease, copper is supposed to directly cause molecular changes in the brain [51,70].

#### 2.2.1. Alzheimer’s Disease

Alzheimer’s disease is characterized by deposition of amyloid protein plaques (extracellular) and neurofibrillary tangles (intracellular) in the brain associated with neurodegeneration. Mutations in the gene for the amyloid precursor protein (APP) have been linked to the inherited Alzheimer’s disease. Thereby, APP is a transmembrane glycoprotein, which contains the amyloid region. By cleavage of APP, amyloid peptide is released and deposited as senile plaque in the brain, which is one main hallmark of the Alzheimer’s disease [42,43]. Copper has been shown to be involved in this context, since co-localization of copper with amyloid proteins of the plaque was detected. APP contains a copper-binding domain, which might have an important control function in the copper pathway and homeostasis [42,43]. In fact, changes in expression of APP also affect cellular copper levels. For instance, APP knock-out mice showed elevated copper levels in brain and liver, indicating that APP is involved in the copper homeostasis [71]. Furthermore, copper is discussed to directly interact with APP. Amyloid–copper complexes have been shown to generate ROS, and in that way, they may increase oxidative stress via Fenton-based chemistry [12,42,61,72]. In experimental studies, APP could reduce Cu(II) to Cu(I) [43,73]. Hence, this electron transfer step might be involved in the formation of ROS.

Besides copper, other essential trace elements such as iron and zinc are also discussed to be involved in the pathology of Alzheimer’s disease. Dyshomeostasis of these elements has been associated with the diseased state. Furthermore, they seem to play a pivotal role in the formation of senile plaques in the brain. Locally elevated levels of iron, zinc and copper within the amyloid plaques have been detected [42,74]. Both iron and copper have been shown to bind to hyperphosphorylated tau proteins in the neurons, which are the primary constitutions of the neurofibrillary tangles [66,75]. Hence, any dyshomeostasis of these elements in particular is discussed to play a decisive role in the pathogenesis of Alzheimer’s disease [12]. Alteration of the elemental distribution within the cells may be considered one mode of action [12,66,76]. At least abnormal cellular copper distribution is observed with local copper accumulation in the extracellular plaques, implying increased copper levels of up to 0.4 mM copper, while the remaining cells and tissues are deficient in copper [66,77].

Novel promising therapeutic strategies focus on restoring elemental homeostasis in Alzheimer’s disease patients. For instance, treatment with clioquinol has been shown to induce redistribution of copper from the extracellular plaques to the copper-deficient cells. Thereby, deposition of amyloid plaque could be reduced, resulting in less oxidative stress [78].

#### 2.2.2. Wilson Disease

Wilson disease is associated with mutations in the gene encoding for the copper transporter ATP7B, greatly affecting the copper metabolism and homeostasis. Severe consequences are, among others, a massive copper overload in the liver (over 20-fold) because of inhibited biliary copper excretion and excess copper in the brain (8-fold). These increased copper accumulations are linked to damage of liver, neurological impairments and mental decline [3,66,67,79]. Even though copper is supposed to be the primary cause for neurotoxic effects associated with Wilson disease, there are also crosslinks to a potentially contributing iron metabolism via the ferroxidase ceruloplasmin [66]. Mitochondria are discussed to be the central target for copper toxicity in Wilson disease. Damages to mitochondria manifested by structural changes have been detected in livers of early-stage diseased patients. In the later phases, the mitochondrial structure is severely damaged and oxidative damage occurs, leading to impaired function [32,67,80].

Medical treatments include application of zinc or chelators such as penicillamine, though this treatment is only effective in early-stage Wilson disease patients. Consequently, early diagnosis and treatment of presymptomatic patients are crucial yet difficult because of variable clinical characteristics [2].

#### 2.2.3. Pending Objectives—Investigation of Key Players

Altered elemental homeostasis (besides copper, as well as zinc and iron [12]) has been clearly associated with neurological disorders, illustrating the importance of copper for physiological brain function [4]. However, the toxic mechanism is not yet fully understood. Especially the context between toxic effects and small changes in copper levels and cellular redistribution on molecular levels is not clear. The molecular basis for copper homeostasis and co-localization with other important proteins and essential elements on the cellular level is one of the major objectives to be investigated. A better comprehension of the toxic mechanisms induced by imbalanced elemental homeostasis would help to understand the role of the involved trace elements as a key player for the brain function and, moreover, the pathological processes of neurological diseases.

Hence, alterations in elemental homeostasis but also redistributions within the cells need to be analyzed on subcellular levels. So far, predominantly total cellular copper levels have been analyzed applying spectrometric methods such as inductively coupled plasma mass spectrometry (ICP-MS) or atomic absorption spectroscopy (AAS). The analysis of cellular elemental distribution within the organelles was mostly limited to cell fractionation followed by ICP-MS analysis [81,82,83]. However, in recent years, high-resolution techniques have been evolved, which provide powerful tools for elemental mapping on the subcellular level.

## 3. Current Research Interests

The trace element copper is essential for biochemical processes. Therefore, the organism developed specific uptake, excretion and translocation mechanisms to regulate a balanced homeostasis. There are still open questions regarding the transport mechanism of copper to the cellular site of action and cellular compartments. In particular, mitochondria play an essential role in the copper homeostasis [33,35]. They are discussed to be one of the major target organelles for copper toxicity and thus might present a crucial part in the pathology of copper-related diseases.

Any disruption of the copper homeostasis can lead to adverse effects and has been associated with human diseases. Altered elemental homeostasis of copper has been linked to neurodegenerative disorders including Menkes and Alzheimer’s disease [70]. Therefore, the research focus is set on elucidation of the underlying toxic mode of action mediated by copper imbalances, with particular focus on neurotoxic effects in the brain [62,63]. In this context, the role of copper and its interplay with other trace elements needs to be elucidated in order to understand the link between elemental imbalances and their roles in pathology. Besides the effects on homeostasis, information about the subcellular localization (e.g., organelles) and changes of element species (e.g., oxidation state and ligand/protein binding) in the cells are of great interest in this context. Furthermore, studies on the co-localization of copper with proteins are an important research area [84]. Subcellular analyses are required to shed light in this context. Cellular bioimaging of copper and relevant elements and biomolecules will provide a fundamental step towards understanding the role of copper in the cells. Highly sensitive and high-resolution techniques are necessary to realize these aims. Based on these research questions, relevant bioimaging techniques will be described and compared in the following paragraphs.

## 4. Analytics of Copper in Cells

### 4.1. Sample Preparation

A fundamental prerequisite for effective bioimaging is well-prepared samples, suitable for the respective method of choice. Without an appropriate sample preparation, high-resolution techniques are worthless. Biological samples pose major challenges for bioimaging analyses, since some analytical techniques need specific requirements (e.g., high vacuum, conductive sample surface) for the measurement, which the native biological samples do not withstand [85,86]. Therefore, biological samples require elaborated sample preparation, whereby the biological structure and chemical composition of the biological samples have to be preserved to circumvent any re-localization or even wash-out of physiologically relevant molecules or metal ions such as copper [87,88,89,90]. Moreover, no artifacts should be formed during this preparation process, which might disturb the measurements [91].

Depending on the imaging technique, basic steps in sample preparation for biological samples include immobilization of the biological structure, dehydration, mounting in resin, and sectioning [88]. By modifying and adapting these steps, an appropriate sample preparation can be acquired for the respective analytical technique, depending on the research focus. For subcellular localization of copper, a major challenge in sample preparation is to avoid any redistribution of copper within the cell.

#### 4.1.1. Fixation

##### Chemical Fixation

In order to minimize biochemical and proteolytic processes and thus, degradation of the biological sample, fixation steps are required. Fixation can be achieved by physical or chemical methods. The most commonly applied chemical fixation is based on the application of fixative agents (e.g., ethanol, methanol, formaldehyde, glutaraldehyde or osmium tetroxide). Alcohol-based solvents lead to protein denaturation and thus stabilize the cellular matrix. However, lipids might be extracted during this procedure. Aldehydes and osmium tetroxide maintain the structural integrity by formation of cross-links between proteins (mainly with lysine via methylene bridges) or unsaturated lipids, respectively, and inhibit enzyme activity [92,93].

Combining several fixative agents has been shown to be more effective. For instance, optimal fixation steps in transmission electron microscopy (TEM) include primary fixation with formaldehyde and glutaraldehyde, followed by secondary fixation with osmium tetroxide, with the additional benefit of improving the contrast for electron microscopy [92]. However, applying a chemical fixation procedure might partly alter the cellular distribution of certain easily diffusible elements. Therefore, this sample preparation should only be considered for elements which tend to tightly bind to larger macromolecules including DNA and proteins or cellular structures like membranes [85,88,94]. In the case of analyses of highly diffusible ions, focus should be laid on physical fixation such as cryogenic sample preparation, which might be better suitable to maintain both structural and chemical integrity [93,95].

##### Cryofixation by Plunge Freezing and High-Pressure Freezing

Cryofixation is based on stopping the cellular activity by shock-freezing the cells at low temperature (−180 °C). Different methods have been developed for cryopreservation including plunge-freezing and high-pressure freezing [88,96].

Plunge-freezing is a simple and widely used way to freeze the sample. Basically, the sample is plunged into liquid cryogen such as propane or nitrogen. High freezing rates minimize the formation of ice crystals, which might destroy cellular structures. The cooling speed depends on the shape and thickness of the sample. In order to obtain optimal freezing conditions, this method is limited to thin samples. For samples thicker than a few micrometers, either pretreatment with cryoprotectants (e.g., 1-hexadecene, glycerol, DMSO) or freezing under high pressure might be required to prevent formation of ice crystals. Bearing in mind that some of these cryoprotectants possess cytotoxic potential at the applied concentrations, non-penetrating cryoprotectants such as sucrose or serum albumin can be applied to circumvent cellular damage [93,97,98].

A special approach, particularly for cell cultures, is freeze-fracturing. Cells are directly cultivated on a silicon wafer, and a second wafer is placed on top like a sandwich. Following plunge-freezing, the two wafers are pulled apart to get two cellular sections, which, however, are not flat and thus might introduce topographic-based effects. This method is typically used for ToF-SIMS (e.g., to study membrane composition) [87,95].

Another approach to achieve ultra-rapid freezing is based on high-pressure freezing. High freezing speed is of crucial importance to maintain the cellular structure. The sample, placed in between carrier plates or sample holder, is pressurized (2100 bar) and rapidly frozen in liquid nitrogen (−196 °C) within milliseconds [97,99]. The application of high pressure shows cryoprotective effects by lowering the melting point and reducing the formation of ice crystals [97].

Since no chemical fixative is used during these physical-based fixation procedures, alterations in elemental distribution are minimized. Thus, this sample preparation seems to be quite applicable for analyses of trace elements (including highly-diffusible elements) in cells using bioimaging techniques such as NanoSIMS [89,93,100].

Subsequently, either the cryofrozen sample can be analyzed in its native frozen state, ensuring that the sample is always processed and kept under −150 °C, or the water needs to be eliminated from the frozen sample using follow-up steps of freeze-substitution or freeze-drying/lyophilization [101,102].

#### 4.1.2. Dehydration

For some techniques, particularly if high vacuum is required, the samples need to be dehydrated before analysis. The easiest way to remove water is the application of solvent baths (e.g., ethanol or acetone). However, regarding the cellular elemental distribution, it is quite likely that re-localization or even leaching occurs during this procedure. Further dehydration methods have been developed, including lyophilization and cryo-substitution.

During lyophilization or freeze-drying, the sample is dehydrated in its frozen state under specific, controlled conditions. Thereby, cellular frozen water sublimates under vacuum. Since no solvents are used to dehydrate the sample by means of lyophilization, changes in local redistribution are minimized [103]. Freeze-dried samples do not necessarily have to be processed and stored under cryogenic temperatures, making their handling easier. However, the cellular structure is most often not as well preserved as compared to freeze-substitution [104].

Within the process of cryo-substitution, frozen water in the cells is replaced by application of organic solvents at low temperatures. Usually, the samples are placed in cryovials in anhydrous acetone at −90 °C for 3 days. Simultaneous fixation can be achieved by adding fixatives such as osmium tetroxide, uranyl acetate and glutaraldehyde. Subsequently, the cryo-substituted samples are allowed to acclimatize to room temperature within several hours and are washed with anhydrous acetone [101]. Combining high-pressure freezing and cryo-substitution is supposed to preserve the integrity of cell membranes and cellular fine structure and thus allows for images of better quality compared to chemical fixation and dehydration techniques [87]. However, there is still a certain risk of wash-out effects due to the substitution procedure [89,97].

#### 4.1.3. Embedding and Sectioning

Embedding the sample in resin followed by sectioning by ultramicrotomy has been shown to provide certain improvements for some analytical techniques. Thereby, the sample is infiltrated by the resin, which is polymerized by heat. There are certain restrictions to the resin, which should show low-degassing properties. Preferably epoxy-based resins are applied [105]. The embedded sample is cut by a diamond knife to thin sections (typically 300 nm for NanoSIMS; 70 nm for TEM/X-EDS) applying ultramicrotomy. The sections are placed on silicon wafers or grids and stored at room temperature until analysis [103,104].

The advantage of resin impregnation and sectioning is that the sample surface is plane, which is particularly important for NanoSIMS analyses to avoid artifacts due to the topography of the sample surface. Moreover, fewer charging effects occur on the sample surface during analysis with an ion beam due to uniform resin matrix [89].

### 4.2. Element- and Molecule-Specific Bioimaging Techniques

Cellular bioimaging provides fundamental information on physiological processes. To visualize the spatial distribution of molecules or element species with high resolution, different techniques (based on fluorescence and X-ray, among other sources) are applied on samples from animal models and tissues, but also in cell culture. Knowledge on the spatial distribution of any target molecule or element of interest, combined with information on localization of other molecules in the chemical environment, is a helpful tool to understand their physiological role and potential interaction. In this context, particularly distinct changes in the subcellular distribution, so-called re-localization effects, are important for the physiological function and its regulation [25,34,106]. Since elemental dyshomeostasis has been associated with neurodegenerative diseases, bioimaging of elements is moreover gaining attraction, because information on the local elemental distribution is important to understand the underlying cellular mechanisms. Up to now, a wide range of bioimaging techniques has been developed and applied. Combining enhanced spatial resolution and high elemental sensitivity with lower limit of detection enables the analysis of samples on the subcellular level down to the nanometer scale [84,100].

Within this sub-section, selected techniques which can be applied for the localization of copper in biological tissue and cells will be described with special focus on their strengths and weaknesses in the field of bioimaging on the cellular level (see Table 1). In general, these techniques are considered rather complementary than competing. Combining the power of different techniques and developing more and more applications enables to gain even more fundamental knowledge and helps to shed light on current research questions. 

#### 4.2.1. LA-ICP-MS (Laser Ablation Inductively Coupled Plasma Mass Spectrometry)

Laser ablation inductively coupled plasma mass spectrometry (LA-ICP-MS) provides a sensitive multi-element analysis technique for bioimaging. Combining ICP-MS with laser ablation enables one to obtain information on both the elemental composition and their spatial distribution [119].

The basic principle is that the surface of a sample is directly ablated and analyzed for its elemental composition. Therefore, a focused laser beam is used to scan over a solid sample, whereby the mass spectrum is continuously analyzed. There is no time-consuming sample preparation required (in contrast to MALDI or NanoSIMS analysis) as 5–100 µm thin cryosections of the biological tissue are directly analyzed [108,119]. The sections are placed on a glass slide and put in the laser ablation chamber. There, the sample is rastered by a pulsed laser consisting of photons under atmospheric pressure, and the released material is analyzed with the inductively coupled plasma mass spectrometer (ICP-MS) (following ionization), which is directly connected with the chamber. A carrier gas (e.g., argon or helium) permanently flows through the chamber, and thus the ablated molecules are transferred to the ICP-MS and quantified regarding their element analysis. For imaging, the relative distribution of an element in the sample is shown by plotting its signal intensities [103,107].

The characteristics of this technique are high sensitivity and very low detection limits (µg–ng/g range) for metals, metalloids and nonmetals. The low detection limits are an outstanding feature of this technique (as compared to X-ray techniques). The cellular distribution of metals or metal-containing molecules can be analyzed with a spatial resolution down to 2–5 µm, allowing this technique to be used for mapping elements in biological tissue [103,108,109,110]. However, analyzing elemental distribution on the cellular or subcellular level is restricted, and special laser optics are necessary to enhance the resolution [103,107].

LA-ICP-MS is not just limited to localization of metals, as other biological molecules can also be investigated by applying an indirect approach. Even though molecular information is lost, as the use of ICP-MS is a destructive method, special applications for LA-ICP-MS have been developed to preserve this information. Thereby, samples are immunologically labeled with antibodies containing lanthanides, which can be detected. In that way, information on the spatial distribution of other molecules (e.g., proteins) can be gained [103,120]. Moreover, combining this mass spectrometry technique with proteomic techniques MALDI/ESI-MS provides a powerful ultra-high-resolution tool to analyze metal-containing biomolecules, for instance, quantification of the phosphorylation of tau proteins in the context of Alzheimer’s disease [107,115].

#### 4.2.2. ToF-SIMS (Time-of-Flight-Secondary Ion Mass Spectrometry)

The basic principle of time-of-flight-secondary ion mass spectrometry (ToF-SIMS) is the sputtering of material from the sample surface by a pulsed primary ion beam. The resulting secondary ions are analyzed for their mass-to-charge ratio using mass spectrometry. The ToF-SIMS can be operated with various primary ion sources, such as Au^+^, Bi^+^ and their respective clusters. The choice of primary ion source determines its destructive power and consequently sputtering characteristics, ion yield and lateral resolution and thus is made with respect to the sample properties and focus of the research aims [105,111,112,121].

In general, this technique provides very high sensitivity (mg–µg/kg range). The ion yield can even be improved by placing the cell sample on a silver surface. Due to the formation of silver cationized species (M+Ag)^+^, higher ion yield is achieved. This approach enables one, for instance, to localize and identify membrane lipids [122].

In general, SIMS analyses can be operated as dynamic or static SIMS, depending on the depth of analysis. While in static SIMS, just the first layers of the surface are analyzed, the dynamic mode characterizes the depth distribution. Usually, ToF-SIMS is predominantly operated as static SIMS [111,123].

Key strengths of ToF-SIMS measurements comprise detection of all secondary ions in parallel in the same polarity and that organic molecules can be analyzed [105]. Thus, this technique is ideal for molecular imaging of complex biological samples, whereas isotope analysis is not feasible due to restricted resolution [88,123].

ToF-SIMS also exhibits some restrictions such as spatial resolution (100–500 nm) [111,112]. Moreover, since ToF-SIMS analysis requires ultra-high-vacuum conditions, an appropriate sample preparation (e.g., cryofixation) is essential. Typically, the samples are cut into 10–20 µm planar sections, although it is possible to analyze frozen, hydrated cells by placing the sample on a cooled stage during measurement [112]. In general, the data handling is a significant challenge because the mass spectra of a biological sample measured by ToF-SIMS contains a huge amount of information on chemical structures. The major challenge is thus to identify the compounds of interest.

#### 4.2.3. TEM/X-EDS (Transmission Electron Microscopy Coupled with Energy-Dispersive X-ray Spectroscopy)

Transmission electron microscopy coupled with energy-dispersive X-ray spectroscopy (TEM/X-EDS) relies on the interaction of a sample with a high-voltage electron beam. Thus, information on sample structure can be gained, and moreover, the emitted characteristic X-rays provide information on the elemental composition [116]. TEM represents a powerful technique to image the cellular structure with a typical resolution down to 0.2 nm. Furthermore, the combination with X-EDS enables elemental mapping with a resolution of 5 nm, however, with relatively low sensitivity (mg/kg range) compared to other techniques (e.g., LA-ICP-MS, NanoSIMS or S-XRF), enabling one to detect major elements but not trace elements. Moreover, this technique provides qualitative rather than quantitative information on the elemental composition [100,103].

Biological samples require an elaborated sample preparation for this technique. Regarding the high-vacuum conditions and high-energy electron beam, the native sample would not be stable and would be unable to withstand this procedure. Moreover, samples need to be thin enough for the electrons to be transmitted through the sample. Usually, optimal sample preparation comprises immobilization and fixation to preserve the cellular structure, dehydration and resin embedding. Finally, thin planar sections, not thicker than 150 nm are prepared and placed on a grid (e.g., Cu, Au). The size of sample can vary from a few nano- to several micrometers [106,116].

In practice, the application of TEM reveals some limitations. With X-ray based techniques, no isotope analysis is available, which would be important to study metabolic processes within the cells [103].

**Figure 2:**
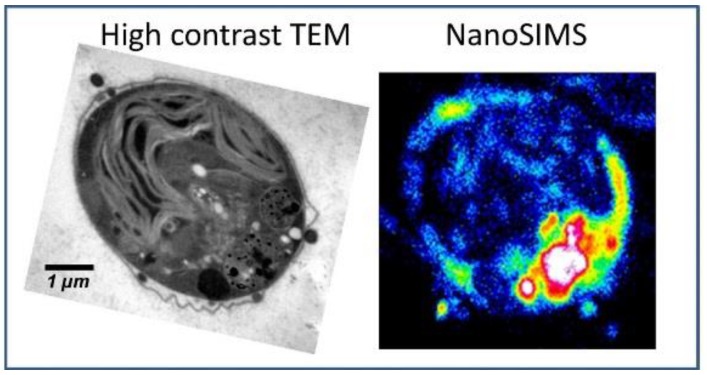
Ultrastructural analysis applying high-contrast TEM combined with elemental distribution applying NanoSIMS. Reprinted from Penen et al.; JTEMB 37, p. 62–68 (2016); with permission from Elsevier [100].

Measurements with TEM can be easily combined with NanoSIMS analysis. Since they both show similar demands on the sample preparations, the same sample can be analyzed applying both techniques. This complementary approach can be used to combine the strengths of both techniques for an improved elemental mapping (see Figure 2). With NanoSIMS, elemental distribution in the cells can be sensitively measured. Thereby, major organelles such as nucleus, membranes and cytoplasm are visualized. TEM contributes with high-contrast analysis and provides structural information on smaller organelles including mitochondria [100,103,124].

#### 4.2.4. Synchrotron-Based XRF (X-ray Fluorescence Spectroscopy)

Synchrotron radiation is generated by high-energy electrons. These electrons are accelerated up to the speed of light under high vacuum and are deflected by magnets. Any bend of the beam results in emission of photons (infrared to hard X-rays). In the case of XRF, the sample is irradiated with X-rays. As a result, atoms release core electrons, which are refilled by outer electrons, and thereby X-ray fluorescence is emitted, resulting in an element-specific spectrum [84,103].

XRF is often combined with XAS (X-ray absorption spectroscopy). Important information can be derived from the absorption spectrum of a sample. In particular, the energy shift and shape of the absorption edge is examined in more detail, which is determined by the state of the analyzed element. Near the absorption edge, XANES (X-ray absorption near-edge structure) is used to identify the oxidation state and obtain information on the coordination environment (e.g., complexes with ligands). The more distant regions reveal information on the chemical environment and coordination parameters (e.g., number and distance) using the extended X-ray absorption fine structure (EXAFS) [115,125,126]. This major analytical strength highlights XRF compared to other commonly used techniques, which are not able to distinguish between different oxidation states or metal species.

In conclusion, by combining these techniques, valuable information on oxidation state or element species, concentration and local distribution can be gained, which is particularly interesting in the context of copper. XRF provides high selectivity and sensitivity (mg–µg/kg range), whereby the sensitivity increases with the element number; thus, it is preferably used for heavy metals. Depending on the synchrotron beamline, imaging can be performed with lateral resolution down to 20 nm and, in case of XAS spectroscopy, down to 50 nm [103]. Typically, samples are analyzed at 50–100 µm sections [117,118,127]. Moreover, this technique offers the possibility to analyze the samples in hydrated state; therefore, no elaborated sample preparation such as resin embedding is required. The sample can be analyzed in its cryogenic state, and thus, any alteration of the native sample is minimized. If the analysis is performed at cryogenic temperatures radiation, damage to the biological sample is also reduced [125].

However, there are certain limitations of this X-ray-based technique, including no potential isotope analysis, which would be important for investigation of the metabolic processes [103].

#### 4.2.5. NanoSIMS (Nano Secondary Ion Mass Spectrometry)

The nano secondary ion mass spectrometry (NanoSIMS) analyses are based on sputtering of the sample surface and thereby creating ions, which are detected by a mass analyzer. In detail, a primary ion beam elutes secondary ions from the solid sample surface. Subsequently, the secondary ions are detected by mass spectrometry. The primary ion beam is either positively (CsCO_3_ source: Cs^+^) or negatively (RF plasma oxygen source: O^−^) charged, depending on the elements of interest. For electronegative elements (e.g., C, N, O, P, S), the cesium source is preferably used, resulting in negative secondary ions. In the case of most metals, the oxygen source is applied and leads to positive secondary ions. Some elements, including copper, arsenic and selenium, can be analyzed with both sources. The two ion sources provide similar spatial resolution (Cs: 50 nm, RF Oxygen: 40 nm) [103,113,114]. Secondary ions are analyzed with a double sector mass analyzer. In parallel, up to seven different ion masses can be detected simultaneously with high mass resolution. The possibility for multi-element analyses combined with outstanding spatial resolution (down to 40–50 nm) makes the NanoSIMS a powerful tool for bioimaging [123,124]. Moreover, elemental mapping with NanoSIMS also includes isotope analysis, which enables one to analyze metabolic processes and the fate of isotope-labeled molecules in the cells [128]. The sensitivity also depends on the analyte of interest and the instrumental conditions (mg–µg/kg range). Generally, NanoSIMS represents the state-of-the-art bioimaging technique to localize trace elements in biological samples by combining its high sensitivity and spatial resolution.

However, there are also some limitations, including high requirements for the sample preparation, so some challenges need to be faced. NanoSIMS operates under high vacuum; therefore, the sample needs to be stable and withstand ultra-high-vacuum conditions. Biological samples must be dehydrated, and the cellular structure needs to be stabilized before analysis. Embedding the sample in resin enhances the stability and minimizes charging effects on the surface of the sample during analysis. For the analysis, thin planar sections of 300–400 nm are prepared. However, the use of resin also increases potential interferences and matrix effects during the measurement. Therefore, quantification is difficult and remains limited due to matrix effects. Taken together, an elaborate sample preparation is required and neither imaging samples in their frozen state nor live-cell imaging is possible [105,114,123].

So far, NanoSIMS has been preferably used for research questions regarding macro-sized biological samples and tissues, including fish, rice and plants [86,105]. However, this technique gains more and more attraction to analyze samples deriving from cell culture as well, because imaging is not just limited to detecting metals in the cells. Cellular structures and biological molecules including lipids can also be visualized [100,128].

### 4.3. Bioimaging Applications with Focus on Copper-Related Diseases

As detailed knowledge about metal homeostasis increased over recent years, more and more biomolecules have been discovered which are involved in transport, trafficking and storage of metals such as copper. Imbalances in the copper homeostasis have been associated with neurological disorders including Alzheimer’s and Wilson disease. However, there are still open questions, particularly with respect to their precise role in pathophysiology of diseases [115]. Thus, with regard to copper-related neurodegenerative diseases, a major research focus is set on the investigation of the cellular impact of copper in regards to cellular distribution and transport of copper under both physiological and diseased state to understand the underlying mechanisms. In this context, especially the role of cellular organelles, interplay with other trace elements and potential co-localization with other biomolecules is elucidated on the subcellular level. Therefore, spatially resolved techniques for elemental mapping have been applied. State-of-the-art bioimaging techniques comprise LA-ICP-MS, SIMS techniques and S-XRF [103].

Neurological disorders have been associated with altered levels of iron, copper and zinc in the brain [74,84]. Thereby, not only were total levels affected, but more interestingly, the spatial distribution was changed. With regard to Alzheimer’s disease, the focus has been on the analysis of the characteristic amyloid plaques associated with this disease. Synchrotron-based analysis revealed that these plaques locally contain high amounts of copper and zinc and slightly increased amounts of iron [115,129]. Thus, it seems apparent that these elements play a role in the formation of amyloid plaques and are connected to the progress of the disease. Early diagnosis of amyloid plaque formation and involved elements might pose a potential way to treat Alzheimer’s disease before symptoms are recognized and neurons have already been affected [115].

Wilson disease is characterized by manifold increased copper levels in liver and brain, which is also taken into consideration as a potential diagnostic tool. However, the distribution of copper has been shown to be rather inhomogeneous. The major amount of the hepatic copper is located in the mitochondria [108,130]. This emphasizes the need to elucidate the role of mitochondria in copper homeostasis and analyze the spatial distribution of the elements rather than total amounts [32].

So far, primarily fluorescence-based probes have been used to detect and localize copper within organelles such as mitochondria. The developed probes selectively sense copper within the mitochondria [131]. However, interferences might disturb the measurement (for instance, quenching effects). Moreover, there is no information on the coordination of copper, e.g., if co-localized with proteins or other biomolecules. Co-localization of copper with proteins has been suggested before applying NanoSIMS in biological samples [86]. Therefore, both, free and bound copper needs to be considered, especially since copper occurs mainly protein bound. Further studies are necessary to investigate and characterize any co-localization of copper with biomolecules. Combining techniques such as LA-ICP-MS and SIMS techniques (e.g., NanoSIMS and ToF-SIMS) would provide important information on the local distribution of metals and biomolecules in parallel, thereby using the respective strengths of spatial resolution (NanoSIMS), low detection limits of metals (LA-ICP-MS) and identification of biomolecules (ToF-SIMS) [107,114]. For instance, LA-ICP-MS, with its lower spatial resolution, could be first used to get an overview, map large areas and select a region of interest in a tissue. Subsequently, SIMS techniques, with their high spatial resolution, provide more detailed analyses on the cellular level. This complementary use of techniques enables improved applications to achieve the research objectives.

In the brain, astrocytes are discussed to be essentially involved in the copper homeostasis [60]. They seem to provide a storage for other brain cells by accumulating large copper amounts. Copper-rich aggregates have been detected in astrocytes isolated from rodent brains, which reveal copper concentration in the millimolar range [53]. For this kind of copper depot, copper-binding proteins such as GSH and MT might be involved and be part of these aggregates. XANES analysis revealed that it is the oxidation state of Cu(I) being present in these aggregates. Even though studies with MT-knockout mice did not show any effects on the overall metal concentration in the brain compared to controls, less aggregates could be detected in the knockout mice, while the copper concentration of the remaining aggregates was still in a similar range as measured by XRF [53]. Therefore, MT is likely to be involved in the formation of these copper-rich aggregates. Their precise role in the copper homeostasis and moreover if there are any potential links to copper-related diseases remain a focus of further investigations.

Interestingly, copper has been discussed to play a role in neuronal signaling in the brain. Perrin and coworkers analyzed the homeostasis of copper in rat neurons applying synchrotron-based XRF imaging. In contrast to zinc, copper was mainly accumulated in the dendrites compared to the cell soma. Within the dendrites, zinc was found to be present all over the dendrites, while copper was localized within certain spots. The authors suggested that both elements are essential for the stabilization of dendrites [132,133]. Future studies should focus more in detail on this link, since this would certainly provide further mechanistic hints about why copper imbalances are associated with neurodegenerative diseases including Alzheimer’s disease.

The copper-binding protein PrP has been shown to be involved in the copper homeostasis in the brain, and its expression correlates with the copper concentration [134]. Apparently, besides regulating the distribution of copper, PrP is likely to possess other characteristics [115]. Synchrotron-based XAS studies characterized the potential antioxidative properties of the copper-binding protein PrP. Copper showed a much higher binding affinity compared to zinc, resulting in redox-inactive coordination of copper, probably resulting in less ROS [135].

## 5. Future Developments and Conclusions

Copper is involved in many cellular processes; consequently, any disruption of the physiological homeostasis likely results in impaired body function and diseased states. Imbalances and subcellular redistribution of copper have been clearly linked to neurodegenerative disorders including Wilson and Alzheimer’s disease [70]. However, the exact mode of action remains the focus of investigations. High-resolution imaging studies, which might characterize elemental distribution, trafficking and co-localization of copper with biomolecules on the cellular level, are scarce. It is of particular importance to uncover the link to the pathological state in this context.

Using high-resolution techniques to investigate effects on the cellular level will provide insights in the molecular and biochemical mechanisms that are involved in neurological diseases. In that way, these studies will elucidate the toxic mode of actions and help to understand the role of copper and other trace elements in the development and pathology of diseases. Based on these investigations, therapeutic strategies can be developed which could help to restore physiological conditions and thereby minimize or even partly prevent copper-related disorders [115].

Several high-resolution bioimaging techniques are available and need to be appropriately selected with regard to the respective biological context. By combining the strengths of these techniques, the yield of valuable information about the sample is enhanced. Thus, analyses may significantly contribute to elucidating relevant research questions regarding copper-related diseases.

In this context, finding adequate biomarkers for the aforementioned disorders is of utmost importance; however, it remains challenging so far. The copper metabolism adapts slowly to changes in dietary copper uptake, making early diagnosis for any alterations difficult [136]. However, diagnosis as soon as cognitive symptoms are recognized is too late for any therapeutic action. Therefore, early biomarkers that are for instance based on the elemental distribution or formation of amyloid plaques (where metals are involved) might be a potential tool to diagnose a disease or monitor its progression. Subsequently, based on these markers, therapeutic strategies (e.g., effective drugs) can be developed to treat or even prevent any disruption.

## Figures and Tables

**Table 1:** Major analytical techniques for bioimaging and their characteristics.

Technique	LA-ICP-MS [103,107,108,109,110]	ToF-SIMS [95,111,112]	NanoSIMS [89,100,103,105,113,114]	TEM/X-EDS [103,115,116]	Synchrotron-Based XRF [103,117,118]
**Beam**	Photons	Ions	Ions	Electrons	X-rays
**Spatial Resolution**	2–5 µm	100–500 nm	40–50 nm	0.2 nm for structural imaging 5 nm for elemental mapping	20 nm
**Sensitivity**	low µg/kg range	mg–µg/kg range	mg–µg/kg range	100–1000 mg/kg	mg–µg/kg range
**Dynamic Range**	10^9^	10^7^	10^2^	-	10^3^
**Advantage**	- simple sample preparation - less matrix effects - no vacuum required - high sample throughput - quantitative analysis - isotope and multi-element analysis (preferably metals) - excellent sensitivity	- highly sensitive - multi-element analysis (detection of all different ions in parallel) - analysis of organic molecules	- high spatial resolution - isotope and multi-element analysis (nonmetals, metalloids, metals)	- structural imaging - more sensitive for metals	- simple sample preparation (can be analyzed in still hydrated and frozen state) - detailed information on oxidation states/element species/ligands - quantitative analysis - more sensitive for metals
**Disadvantage**	- lower spatial resolution - mainly limited to metals - destructive analysis	- charge compensation required for insulated biological samples	- elaborated sample preparation - needs high-vacuum conditions - no live-cell analysis - no quantification due to large matrix effects - destructive analysis	- needs high-vacuum conditions - lower sensitivity - qualitative rather than quantitative analysis	- no isotope analysis
**Preferred Sample Characteristics**	biological tissue or planar sections (5–100 µm) sample can be hydrated and frozen (use of cryogenic cell)	planar sections (10–20 µm) sample can be hydrated and frozen (use of cryostage)	thin, planar sections (300–400 nm)	thin, planar sections (70–150 nm)	planar sections (50–100 µm) sample can be hydrated and frozen (use of cryostage)
**Sample Preparation**	- frozen and hydrated sample is placed on grids but can also be embedded in resin	- fixated sample is typically placed on grids/sample holder - cryofixation or freeze-fracturing - metal coating (e.g., Au, Pt, Ag) may be necessary to avoid charging effects	- dehydrated and fixated sample is typically placed on wafers - chemical fixation/dehydration/resin embedding - cryofixation/freeze-substitution/freeze-drying/resin embedding - metal coating (e.g., Au, Pt, Ag) may be necessary to avoid charging effects	- dehydrated and fixated sample is typically placed on grids - chemical fixation/dehydration/resin embedding - cryofixation/freeze-substitution/freeze-drying/resin embedding	- frozen and hydrated sample is placed on grids/sample holder

## Abbreviations

AAS	atomic absorption spectroscopy
APP	amyloid precursor protein
ATOX-1	human antioxidant protein-1
BBB	blood-brain barrier
BLB	blood-liquor barrier
CCS	copper chaperone for superoxide dismutase 1
COX17	copper chaperone for cytochrome c-oxidase
CSF	cerebrospinal fluid
Ctr1	copper transporter receptor 1
Ctr2	copper transporter receptor 2
DMT1	divalent metal ion transporter
EXAFS	extended X-ray absorption fine structure
GSH	glutathione
ICP-MS	inductively coupled plasma mass spectrometry
LA-ICP-MS	laser ablation inductively coupled plasma mass spectrometry
MT	metallothionein
NanoSIMS	nano secondary ion mass spectrometry
PrP	prion protein
ROS	reactive oxygen species
S-XRF	synchrotron-based X-ray fluorescence spectroscopy
TEM	transmission electron microscopy
ToF-SIMS	time-of-flight-secondary ion mass spectrometry
XANES	X-ray absorption near-edge structure
XAS	X-ray absorption spectroscopy
X-EDS	energy-dispersive X-ray spectroscopy
